# Assessment of the diagnostic performance of serological tests in areas where *Leishmania infantum* and *Leishmania tarentolae* occur in sympatry

**DOI:** 10.1186/s13071-023-05981-0

**Published:** 2023-10-08

**Authors:** Roberta Iatta, Mariaelisa Carbonara, Anna Morea, Paolo Trerotoli, Giovanni Benelli, Yaarit Nachum-Biala, Jairo Alfonso Mendoza-Roldan, Maria Alfonsa Cavalera, Gad Baneth, Claudio Bandi, Andrea Zatelli, Domenico Otranto

**Affiliations:** 1 Interdisciplinary Department of Medicine, University of Bari, Bari, Italy; 2 Department of Veterinary Medicine, University of Bari Aldo Moro, Valenzano, Italy; 3 Department of Agriculture, Food and Environment, University of Pisa, Pisa, Italy; 4 School of Veterinary Medicine, The Hebrew University of Jerusalem, Rehovot, Israel; 5 Department of Biosciences, Pediatric CRC “Romeo Ed Enrica Invernizzi”-University of Milan, Milan, Italy; 6 Department of Pathobiology, Faculty of Veterinary Science, Bu-Ali Sina University, Hamedan, Iran

**Keywords:** Canine leishmaniosis, ELISA, IFAT, *Leishmania* spp., Performance, Serological tests

## Abstract

**Background:**

Visceral leishmaniosis caused by infection with the zoonotic protozoan *Leishmania infantum* is a life-threatening disease affecting dogs and humans. The sympatric occurrence of *L. infantum* and *Leishmania tarentolae* in an area of southern Italy endemic for canine leishmaniosis, where dogs are also exposed to the latter species, suggests the persistence of herpetophilic *L. tarentolae* in a non-permissive host, therefore raising questions about the performance of serological diagnostic tests routinely employed.

**Methods:**

The diagnostic performance of serological tests such as the immunofluorescence antibody test (IFAT), two commercial immunoenzymatic assays (i.e. NovaTec VetLine Leishmania ELISA® and rK39 ICT®) and an in-house enzyme-linked immunosorbent assay (ELISA) was evaluated in healthy dogs seropositive to *L. infantum*, whereas the only IFAT available was used to detect antibodies to *L. tarentolae*.

**Results:**

With the IFAT, out of a total of 104 dogs tested, 15 were seronegative for *L. infantum* of which three were *L. tarentolae* seropositive‚ and 89 were *L. infantum* seropositive. Of the latter 89 dogs, representing the highest proportion of seropositive animals (85.6%) detected by IFAT‚ 66 were also seropositive for *L. tarentolae*. Cohen's kappa (*κ*) agreement coefficient between the IFAT results and those of all the other tests was very low, and the IFAT results were significantly different from those of all the other serological tests as calculated by Cochran's Q-test. Analysis using the Bayesian latent class (Bayes-LCA) showed that the in-house ELISA and IFAT contributed the most towards identifying infected and non-infected dogs, respectively. The IFAT test showed low positive predictive value (59.5%), but high negative predictive value (100%).

**Conclusions:**

These results demonstrate that the IFAT for *L. infantum*, although highly sensitive, may not be considered a useful diagnostic test due to its low specificity. Therefore, an accurate serological tool with high specificity is mandatory for avoiding cross-reaction in epidemiological contexts where the two species of *Leishmania* occur in sympatry*.*

**Graphical Abstract:**

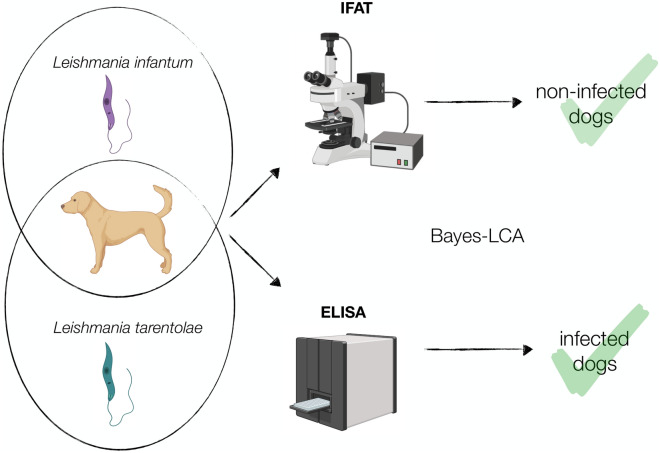

## Background

Visceral leishmaniases (VL) are life-threatening diseases caused by the anthropic *Leishmania donovani* in East Africa and India and the zoonotic *Leishmania infantum*, which is widely distributed in the Middle East and Central Asia, across the Mediterranean basin and in Latin America [1]. These flagellated protozoa are transmitted by female phlebotomine sand flies (Diptera, Psychodidae) that feed on the blood of a vertebrate host [2]. Dogs play a crucial role in the maintenance of the zoonotic visceral form as they are the primary reservoirs of the parasite [3]. Infection by *L. infantum* may remain asymptomatic or evolve towards overt clinical disease, depending on the immune response of infected animals [4]. Therefore, the diagnosis of leishmaniosis is relevant both for diagnosing clinical forms in veterinary practice as well as for investigating the epidemiology of the infection, with the ultimate aim of implementing control measures.

Currently, the laboratory approach for an etiologic diagnosis in dogs with clinical manifestations or subclinical infections is based on serology for the detection of antibodies (indirect tests) and/or detection of the parasite or its components (direct tests) [5]. The most suitable assay used for detecting antileishmanial antibodies is the immunofluorescence antibody test (IFAT), as recommended by the World Organization for Animal Health [6]. Enzyme-linked immunosorbent assays (ELISA) and immunochromatographic tests (ICT) are also employed for the serological diagnosis of canine leishmaniosis (CanL). All of these tests are usually characterized by high sensitivity (Se, though rarely of 100%), but cross-reaction with antibodies against other species of *Leishmania* or other trypanosomatids, such as *Trypanosoma cruzi*, may impair their specificity (Sp) [7, 8]. Importantly, the accuracy of serological assays may change depending on the use of crude soluble antigens or single/multiple recombinant protein antigens [8]. Conversely, parasitological tests based on microscopic observation of *Leishmania* parasites or the isolation in culture media have a high Sp (around 100%), but low Se [9]. Molecular tests are accurate, but their performance depends on the biological samples analyzed, the target genes and the PCR methodology employed, with the quantitative PCR of the kinetoplast DNA minicircle (kDNA) among the most frequently performed.

Interestingly, in a CanL endemic area of southern Italy where reptiles, herpetophilic sand flies and dogs share the same environment, the sympatric occurrence of *L. infantum* and *Leishmania tarentolae*, with the latter for a long time considered to be a trypanosomatid associated exclusively to saurians, has been reported [10]. In this context, dogs have been found that were seropositive for *L. tarentolae*, with some of them remaining seropositive even during the non-transmission sand fly season [10]. This persistence of seropositivity suggested that infection by *L. tarentolae* in these dogs was intense enough to determine a detectable and rather persistent immune response, and was not just a transient presence of the protozoan in a non-permissive host [10]. Furthermore, the detection of *L. tarentolae* DNA in lizards and shelter dogs raised questions about the effects that its exposure may have in dogs [10]. Accordingly, the occurrence of *L. tarentolae* has been recognized as an opportunity for stimulating the cellular responsiveness of exposed animals against other species, but also a hindrance causing possible serological cross-reaction [10, 11].

In addition, the detection of *L. tarentolae* DNA in geckoes, in *Sergentomyia minuta* [12, 13] and the sand flies *Phlebotomus perfiliewi* and *Phlebotomus perniciosus*, although unusual as both of the latter species are vectors of *L. infantum* [14–16] and normally feed on mammalian blood, supports the findings of *L. tarentolae* DNA in human subjects in southern Italy [15, 17]. While the vector competence of these phlebotomine sand flies in transmitting *L. tarentolae* to mammals is still unclear, potential health implications should be considered in epidemiological contexts where *L. infantum* and *L. tarentolae* occur in sympatry.

To investigate further the diagnostic performance of routinely used serological tests in areas where dogs have been shown to be seropositive for both *L. infantum* and *L. tarentolae* by IFAT [10, 17], we tested apparently healthy dogs using IFAT together with three additional tests, namely two commercially available serologic tests (i.e. NovaTec VetLine Leishmania ELISA® and the Kalazar Detect Rapid Test) and an in-house ELISA, for the detection of antibodies against *L. infantum.*

## Methods

### Study population and sample collection

From February 2020 to May 2022, a total of 104 dogs of different sex, age and breed that had previously been clinically evaluated in concluded [18] or still ongoing (data unpublished) trials, which presented no apparent clinical or laboratory signs compatible with CanL, were retrospectively selected for inclusion in the present study based on established criteria. Dogs from two municipal shelters in southern Italy (Lecce: 40.419326N, 18.165582E; Casarano: 40.0126N, 18.1606E) were sampled for blood. Dogs tested for the detection of antibodies against *L. infantum* by IFAT were included [19], while animals vaccinated for leishmaniosis and/or seropositive by IFAT to *Ehrlichia canis* (Biopronix Agrolabo, Scarmagno, Italy) and *Anaplasma phagocytophilum* (MegaCor Diagnostik, Horbranz, Austria) were excluded.

Based on the above-mentioned criteria, the animals were subdivided into two groups according to the IFAT results on *L. infantum* seropositivity, with one group (group A) including those dogs that tested seronegative (i.e. antibody titre < 1:80) and the second group (group B) including those dogs that tested seropositive (i.e. 1:80 ≤ antibody titre < 1:2560).

All serum samples were tested for anti-*L. infantum* antibodies by two commercial serologic tests and an in-house ELISA (detailed below), whereas antibodies anti*-L. tarentolae* were assessed by IFAT as described by Iatta et al. [17]. Samples were considered to be positive by IFAT when they produced a clear cytoplasmic and membrane fluorescence of promastigotes from a cut-off dilution of 1:80. The presence of DNA of both *Leishmania* spp. was also evaluated by real time-PCR of dog blood.

### Serological testing

Serum samples from all enrolled dogs were tested for *L. infantum* antibodies by two commercial serologic tests, namely the NovaTec VetLine Leishmania ELISA® (NovaTec Immundiagnostica GmbH, Dietzenbach, Germany) and the Kalazar Detect Rapid Test (rK39 ICT®; InBios International Inc., Seattle, WA, USA), and by an in-house ELISA.

Commercial assays were carried out according to the manufacturer’s instructions. Briefly, for the NovaTec VetLine Leishmania ELISA®, 100 μl of serum sample diluted 1:100 in the buffer supplied by the kit was added to each microwell coated with *Leishmania* antigens and incubated for 1 h at 37 °C. This was followed by washing and then by a second incubation for 30 min at room temperature with 100 μl of peroxidase-labeled protein A/G conjugate. After washing, 100 μl of 3,3′,5,5′-tetramethylbenzidine solution was added to the wells and the microplate incubated for 15 min at room temperature in the dark; finally the reaction was blocked with sulfuric acid (0.2 mol/l). The absorbance was measured in a microplate reader (model 680; Bio-Rad Laboratories, Hercules, CA, USA) at 450 nm. The test Se and Sp are 95.80% and 95.43%, respectively. Antibodies reactive with the recombinant K39 antigen were tested using the Kalazar Detect dipstick kit (i.e., rK39 ICT) according to the manufacturer's instruction.

Finally, canine sera were tested by an in-house ELISA that contains crude leishmanial antigen. A 100-μl sample of each serum diluted to 1:100 was added in the microplate and incubated for 1 h at 37 °C. The plates were then washed with 0.1% Tween 20 in 50 mM phosphate-buffered saline (PBS), pH 7.2, and incubated with protein A conjugated to horseradish peroxidase (1:10,000 dilution; Zymed Laboratories, Inc., San Francisco, CA, USA) for 1 h at 37 °C. Excess conjugate was removed by washing in PBS-Tween, and the plates were developed by adding the substrate 2,2’-azino-di-3-ethylbenzothiazoline sulfonate (ABTS) (Boehringer Mannheim, Mannheim, Germany). Each plate was read at 405 nm when the absorbance of the positive canine reference serum reached a value between 1.1 and 1.2. A titration of positive and negative reference canine sera was included on each plate to monitor inter-assay variation.

### Molecular testing

Genomic DNA (gDNA) was extracted from canine blood samples by a commercial GenUPBlood DNA kit (Biotechrabbit GmbH, Hennigsdorf, Germany) according to the manufacturer’s instructions. All samples were tested by duplex real time PCR (dqPCR) for the detection of a partial region of the internal transcribed spacer 1 (ITS1) locus of *L. infantum* and *L. tarentolae,* and of *L. infantum* kDNA minicircle (120 bp) by real time-PCR (qPCR), following previously described protocols [20, 21]. Genomic DNA from a *L. infantum* isolate from a dog with leishmaniosis from Italy (zymodeme MON-1) and *L. tarentolae* (strain RTAR/IT/81/ISS21-G.6c/LEM124) promastigotes were used as positive controls, whereas gDNA extracted from blood of a healthy dog and negative for *L. infantum* was used as negative control.

### Statistical analysis

The results were reported as counts and percentages. The homogeneity of positive responses for tests was assessed using Cochran's Q-test, followed by multiplicity-adjusted post-hoc comparisons [22]. For the post-hoc comparison, the least statistically significant difference between two percentages was 18.2%; over that threshold, the comparison between two tests was considered to be statistically significant. The percentages of agreement between test pairs were also determined by the Cohen's kappa (*κ*) agreement coefficient, with *κ* ≤ 0 indicating no agreement; *κ* = 0.01–0.20, slight agreement; *κ* = 0.21–0.40, fair agreement; *κ* = 0.41–0.60, moderate agreement; *κ* = 0.61–0.8, substantial agreement; and *κ* > 0.81, almost perfect agreement. Analyses were done using GraphPad Prism version 8.0.0 (MedCalc Statistical Software version 16.2.1; MedCalc Software Ltd, Ostend, Belgium).

The evaluation of the test’s accuracy was carried out in the absence of a gold standard. Consequently, the assignment of the "infected" or "not infected" class was determined by applying a Bayesian model for the analysis of the latent classes [23]. To this end, a cross-validation process with 10 resamplings was applied, dividing the database into 70% and 30%. The first 70% is the training set, used to apply the model to latent classes, and the second 30% is the validation set for evaluating the test’s accuracy after assigning the classes.

The Bayesian latent class analysis (Bayes-LCA) was applied to the training set, with the variational Bayesian (VB) as the chosen model. The chosen model was better than the Estimation-Maximization or Gibbs sampling estimates when both the deviance information criterion (DIC) and Akaike information criterion (AIC) indexes and the posterior standard deviation (PSD) were calculated, being lower in the Bayes-LCA-VB model than in the other models. The parameters obtained by the Bayes-LCA-VB were applied to the validation sets and used for the determination of the Se, Sp, positive predictive value (PPV) and negative predictive value (NPV). The results are shown as the median and range of the results obtained from the 10 resamplings.

The analysis was conducted using the R software (version 4.2.2) for the creation of the training and validation sets, and the Bayes-LCA package, applying the VB method, for the measurement estimation of accuracy.

## Results

**Fig. 1:**
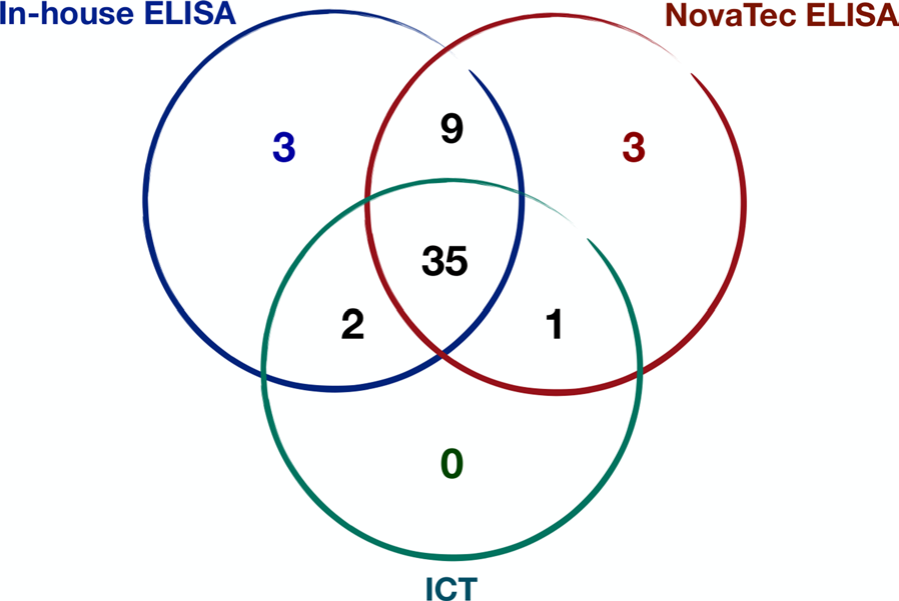
Number of dogs seropositive for *Leishmania infantum* based on the results from the combination of different diagnostic tests among the 53 dogs whose serum samples tested seropositive to both *Leishmania* spp. by the immunofluorescence antibody test. ELISA, Enzyme-linked immunosorbent assay; ICT, immunochromatographic test

All dogs (*n* = 104; 55 [52%] females) were of mixed breed and ranged in age from 4 to 15 (median 6.8) years. Of the 104 dogs, 15 were included in group A (i.e. seronegative for *L. infantum* by the IFAT and all of the other tests), of which 12 were seronegative and three seropositive for *L. tarentolae*, and 89 were included in group B (i.e. seropositive for *L. infantum*), of which 66 and 23 were seropositive and seronegative, for *L. tarentolae‚* respectively. In particular, of the 66 dogs in group B that were seropositive for *L. tarentolae,* 53 tested positive for *L. infantum* by at least one other test (Fig. 1), and the remaining 13 were negative by all of the tests.

**Fig. 2:**
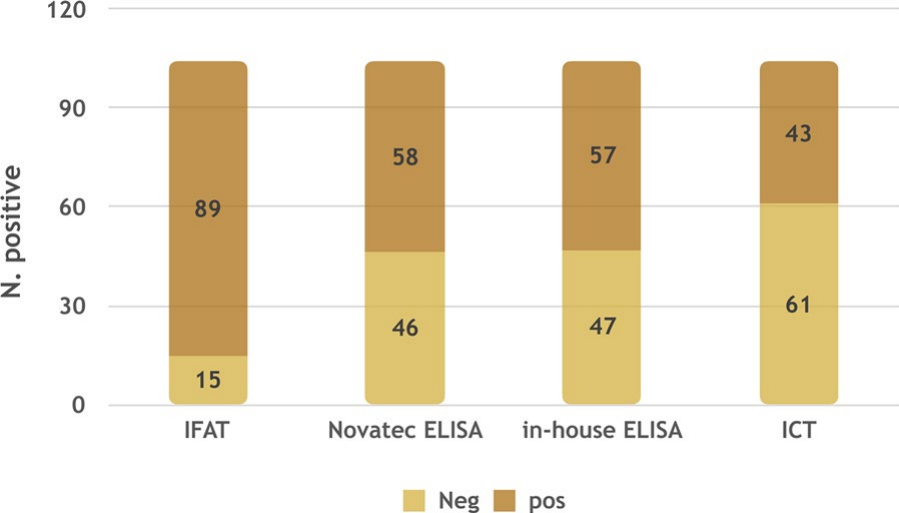
Number of positive and negative serum samples for *L. infantum* by different serological tests. ELISA, Enzyme-linked immunosorbent assay; ICT, immunochromatographic test; IFAT, immunofluorescence antibody test

In addition, 10 out of the 23 dogs in group B‚ which were seronegative for *L. tarentolae*‚ were negative for *L. infantum* by the other tests. The results of all serological tests for the detection of antibodies against *L. infantum* are shown in Fig. 2.

Circulating *L. infantum* kDNA was detected in the blood of 10 dogs (8.6%) seropositive for both *L. infantum* and *L. tarentolae* with immunoglobulin G (IgG) titers > 1:320 and 1:160, respectively. These dogs showed simultaneous seropositivity for *L. infantum* by all three serological tests (NovaTec VetLine Leishmania ELISA®, rK39 ICT and in-house ELISA), with the exception of one dog that was seronegative only by the ICT. Of these 10 dogs, four tested positive also to *L. infantum* ITS1 by dpPCR. By the molecular methods, all blood samples were negative for *L. tarentolae.*

According to Cochran's Q-test, seropositive results could not be considered equal among tests. The post-hoc test revealed that test results from PCR and IFAT were significantly different from those of all the other tests, while the results among the ICT, in-house ELISA and NovaTec VetLine Leishmania ELISA® showed homogeneity.

Cohen's *κ* agreement coefficient (Table 1) revealed a substantial agreement between the results of the NovaTec VetLine Leishmania ELISA® and those of the in-house ELISA, with *k* = 0.61 (95% confidence interval [CI] 0.46–0.76), and a fair agreement between the results of the IFAT and those of the ICT, with *k* = 0.21 (95% CI 0.11–0.32). The IFAT showed the lowest *k* values with all the other tests.

**Table 1:** Cohen's *κ* agreement coefficient and relative 95% confidence intervals for assessing agreement between tests

Serological test	IFAT	NovaTech ELISA	in-house ELISA
NovaTec ELISA	0.29 (0.15–0.44)		
in-house ELISA	0.34 (0.19–0.48)	0.61 (0.46–0.76)	
ICT	0.21 (0.11–0.32)	0.54 (0.39–0.69)	0.58 (0.43–0.73)

Values in table are presented as Cohen's *κ* agreement coefficient with the 95% confidence interval in parentheses. *κ* = 0.21–0.40, fair agreement; *κ* = 0.41–0.60, moderate agreement; *κ* = 0.61–0.8, substantial agreement

*ELISA* Enzyme-linked immunosorbent assay, *ICT* immunochromatographic test,* IFAT* immunofluorescence antibody test

The test accuracy performed excluding the PCR results, showed that all serological tests were simultaneously positive in 34.6% of cases (36/104) as well as simultaneously negative in 13.5% of cases (14/104) (Table 2).

**Table 2:** Frequency distribution of dogs based on the results of the combination of tests (*N* = 104 samples)

Serological test results				Positive
IFAT	NovaTec ELISA	in-house ELISA	ICT	*N* (%)
** + **	+	+	+	36 (34.6)
** + **	−	−	−	21 (20.2)
** + **	+	+	−	11 (10.6)
** + **	+	−	−	7 (6.7)
** + **	−	+	−	7 (6.7)
** + **	−	+	+	3 (2.9)
** + **	+	−	+	2 (1.9)
** + **	−	−	+	2 (1.9)
**-**	+	−	−	1 (1.0)

*ELISA* Enzyme-linked immunosorbent assay, *ICT* immunochromatographic test,* IFAT* immunofluorescence antibody test

The estimated probability for dogs to be infected or not, calculated through the Bayes–LCA, is shown in Table 3.

**Table 3:** The estimated probability for dogs to be infected (class 1) if the test is positive or "not infected" (class 2) if the test is negative calculated through the Bayesian latent class analysis

Serological test	Class 1–infected: 0.55 (0.06)	Class 2—not infected: 0.45 (0.06)
IFAT	1	0.7155
NovaTec ELISA	0.884	0.183
in-house ELISA	0.9255	0.1445
ICT	0.7635	0.0425

*ELISA* Enzyme-linked immunosorbent assay, *ICT* immunochromatographic test,* IFAT* immunofluorescence antibody test

The prevalence of being infected reported as the median of the results obtained from resampling was 0.55 (PSD = 0.06). The in-house ELISA contributed the best towards identifying positive subjects, with the median probability of being "infected" of 0.92 (PSD 0.02). Conversely, the IFAT contributed the best towards identifying a “non-infected” dog, with a probability of 0.72 (PSD 0.07). The median values and ranges of Se, Sp, PPV and NPV results of the validation sets are reported in Table 4**,** which shows that the highest Se (100%) and the lowest Sp (29.2%) and the lowest PPV (59.5%) and the highest NPV (100%) were for IFAT.

**Table 4:** Median values and ranges of sensitivity, specificity and positive and negative predictive values obtained from the validation sets

Serological test	Sensitivity (%)	Specificity (%)	PPV (%)	NPV (%)
IFAT	100 (100–100)	29.2 (13.6–45.4)	59.5 (48.7–71.4)	100 (100–100)
NovaTec ELISA	93.3 (86.4–100)	83.3 (73.9–95.45)	85.2 (73.1–96.6)	92 (86.4–100)
in-house ELISA	96.2 (90.5–100)	86.2 (76.9–100)	88 (76–100)	95.4 (89.5–100)
ICT	80 (64–96)	96 (89.5–100)	95.2 (88.9–100)	82.1 (68–96)

*ELISA* Enzyme-linked immunosorbent assay, *ICT* immunochromatographic test,* IFAT* immunofluorescence antibody test,* NPV* negative predictive value,* PPV* positive predictive value

## Discussion

In the study reported here we assessed the diagnostic performance of IFAT for the detection of anti-*L. infantum* antibodies in dogs living in a CanL endemic area, where *L*. *tarentolae* also occurs. In the absence of a gold standard, the results of commercial serological tests and an in-house ELISA were compared by using a Bayesian approach.

Although IFAT revealed the highest proportion of seropositive animals (85.6%), the agreement between the IFAT results and those of all the other tests was found to be very low. This finding along with the statistical high differences in results among tests found by applying Cochran's Q-test suggest that IFAT has a lower specificity than the other serological assays. Therefore, although IFAT is highly sensitive, it may be not considered a useful diagnostic test in epidemiological contexts where different *Leishmania* spp. occur due to its low specificity. These data are not surprising since cross-reactivity of IFAT was reported in dogs from Brazil where *L. infantum* and *Leishmania braziliensis* infections are endemic [24]. Furthermore, in Israel, sera from dogs infected by *Leishmania major*, *Leishmania tropica* and *L. infantum* were found to be reactive by ELISA, with crude promastigote antigen not being distinctive between *Leishmania* spp. [25].

Conversely, the low proportion of dogs found to be seropositive for *L. infantum* by molecular methods and the results obtained by Cochran's Q-test were expected since blood is not the ideal tissue for molecular diagnosis of *L. infantum* infection due to the low parasite load in blood [26]. The detection of only *L. infantum* kDNA in 10 dogs that tested seropositive for both *Leishmania* spp. by IFAT and by the other three serological tests, with the exception of one dog that tested seronegative by ICT, may indicate the occurrence of a cross-reaction of *L. tarentolae* IFAT*.* Similarly, the fact that 13 out of 66 dogs (group B) were seropositive for both *Leishmania* spp. by IFAT, but negative by all the other tests, may suggest the possibility of false positive results of the *L. infantum* IFAT. Moreover, considering the remaining 53 dogs (group B), the different results of the three diagnostic tests (i.e. 6 seropositive by a single test and 12 seropositive by two tests) suggest a potential cross-reaction with antibodies to *L. tarentolae*. In this scenario the immune response of dogs to the infection by a single or by both *Leishmania* spp. may affect the diagnostic test results. Interestingly, the finding of seropositivity of three dogs to *L. tarentolae* only, with antibody titers of 1:80 and seronegativity to *L. infantum* by all the other tests, suggests that these dogs were exposed to *L. tarentolae*, a species which has been largely ignored by the scientific community and considered to be a non-pathogenic saurian-associated trypanosomatid. Recently, the persistent presence of *L. tarentolae* in dogs, considered non-permissive hosts, was reported in CanL endemic areas where reptiles, *S. minuta* (i.e. an herpetophilic sand fly species) and dogs share the same environment [10]. Furthermore, *L. tarentolae* DNA has been detected in dogs as well as in *P. perniciosus*, a sand fly species which usually feeds on dogs, strongly corroborating the possibility that dogs can be infected by this species of *Leishmania* and that a humoral immune response against the parasite may occur [10].

The performance of the serological tests evaluated herein were further confirmed by the Bayes-LCA, which showed that the IFAT was the best serological test for estimating the probability of dogs to be non-infected and the in-house ELISA was the best test for identifying infected animals. Indeed, the IFAT was more sensitive than the ELISA, with limitations in Sp observed in seropositive dogs from CanL non-endemic area and in dogs seropositive to other pathogens, such as *Anaplasma phagocytophilum*, *E. canis* and *Rickettsia conorii* [7, 19]. In the canine population herein screened, the possibility of cross-reaction by IFAT was excluded since all dogs were serological negative to *A. phagocytophilum* and *Ehrlichia canis.* In addition, the ELISA was found to be the better test for diagnosing clinical leishmaniosis when compared with IFAT.

The differences in the accuracy of the ELISA tests and the ICT, as evaluated in the present study, depend on the use of crude soluble antigens or single/multiple recombinant proteins. The ICT was found to be the most specific since it contains a single recombinant protein (i.e. rK39), and NovaTec VetLine Leishmania ELISA® and the in-house ELISA were found to be the most sensitive. Indeed, qualitative rapid tests, which are easy to perform and interpret, are ideal tools in the clinical practice [7, 27]. Therefore, the choice of serological tools may be based on different settings, including sero-epidemiological screening for determining the exposure of dogs in a geographical region or in surveillance programs, for clinical diagnosis and therapeutic purposes.

In summary, data from published studies clearly indicate that there is an overlapping circulation of *L. infantum* and *L. tarentolae* in “non-natural” hosts and vectors in areas of southern Italy. These non-natural hosts and vectors could therefore play a role in the epidemiological cycle of both protozoa. In this context, the serological cross-reactivity between the two species of *Leishmania* studied here may have important implications in the clinical diagnosis of CanL and, consequently, for the management and treatment of seropositive healthy dogs. Nonetheless, the absence of serum samples from dogs infected by other *Leishmania* spp., such as *L. tarentolae*, represents a limitation of the study that should be overcome in future studies.

Moreover, in an era characterized by ecological and anthropic drivers, such as climate change, urbanization, animal translocation, wildlife movement, international travels and migrations [28–31], the risk of introduction of alien *Leishmania* spp. and the spreading of sand fly populations into new geographical areas should be taken into account.

## Conclusions

Overall, the results of the present study highlight that the IFAT, commonly employed for the serodiagnosis of *L. infantum* infection in dogs, may be not considered an useful test in epidemiological contexts where the two species of *Leishmania* coexist due to its low specificity. Therefore, future studies focused on the standardization of a highly accurate test for the detection of antibodies against *L. tarentolae* are mandatory, as well as studies for assessing the prevalence of *L. tarentolae* infection in dogs and its possible interactions with *L. infantum* in areas where they are sympatric.

## Data Availability

All data supporting the findings of this study are available within the paper.

## Abbreviations

ABTS: 2,2’-Azino-di-3-ethylbenzothiazoline sulfonate
Bayes-LCA: Bayesian latent class analysis
BLAST: Basic local alignment search tool
CanL: Canine leishmaniosis
dqPCR: Duplex real time PCR
ELISA: Enzyme linked immunosorbent assay
ICT: Immunochromatographic test
IFAT: Immunofluorescence antibody test
ITS1: Internal transcribed spacer 1
kDNA: Kinetoplast deoxyribonucleic acid
NPV: Negative predictive value
PBS: Phosphate-buffered saline
PPV: Positive predictive value
rK39: Recombinant K39
Se: Sensitivity
Sp: Specificity
TMB: Tetramethylbenzidine
VB: Variational Bayesian
